# Supplementary figures and images for: Three Seinura species from Japan with a description of S. shigaensis n. sp. (Tylenchomorpha: Aphelenchoididae)
Source: PLoS One. 2021 Jan 6;16(1):e0244653. doi: 10.1371/journal.pone.0244653 (PMC7787460; doi:10.1371/journal.pone.0244653)

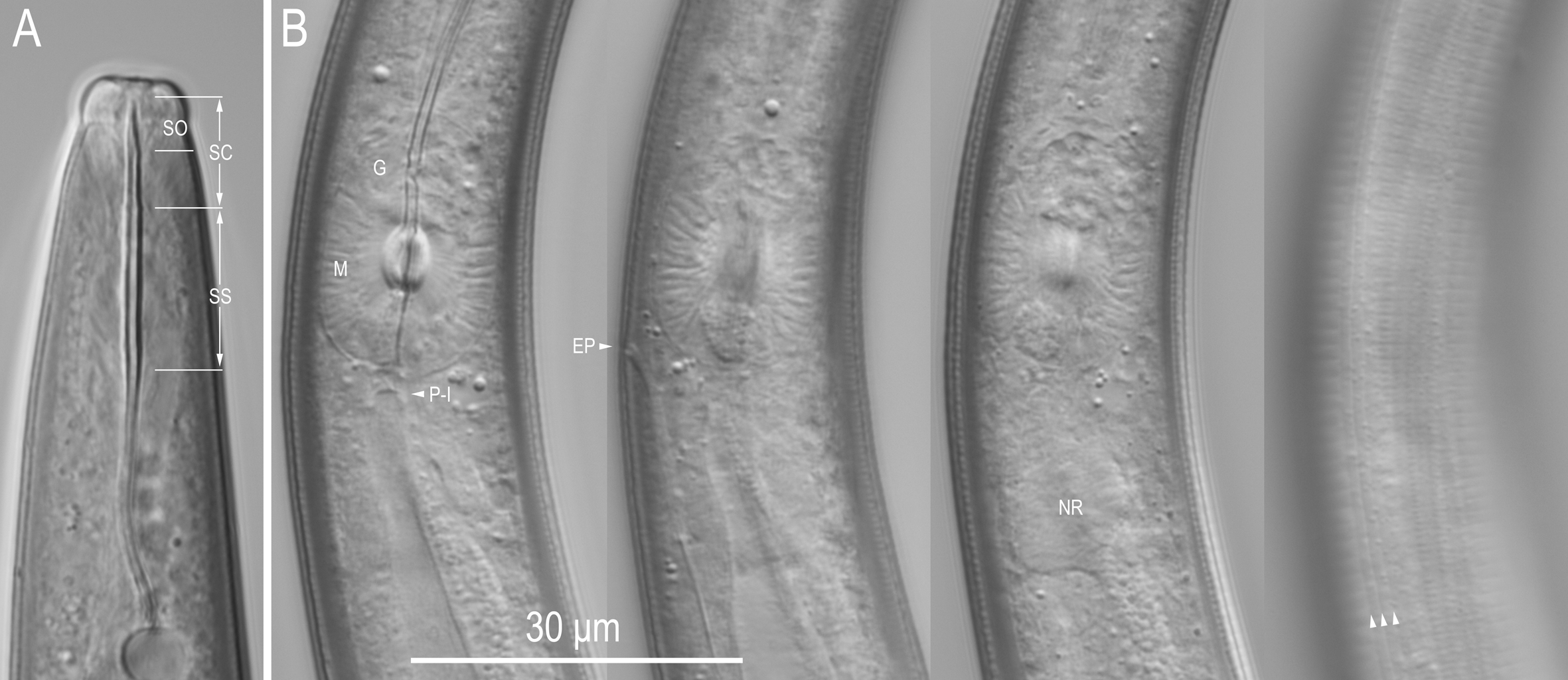

Supplement: S1 Fig — A: Lip and stylet region; B: Metacorpal region in three different focal planes. Abbreviations are as follows: SO = stylet opening; SC = stylet conus; SS = stylet shaft; EP = secretory-excretory pore; G: glandular part of median bulb; M = muscular part of median bulb; P-I = pharyngo-intestinal junction; NR = nerve ring; arrowheads = lateral lines. (TIF) [file pone.0244653.s001.tif]

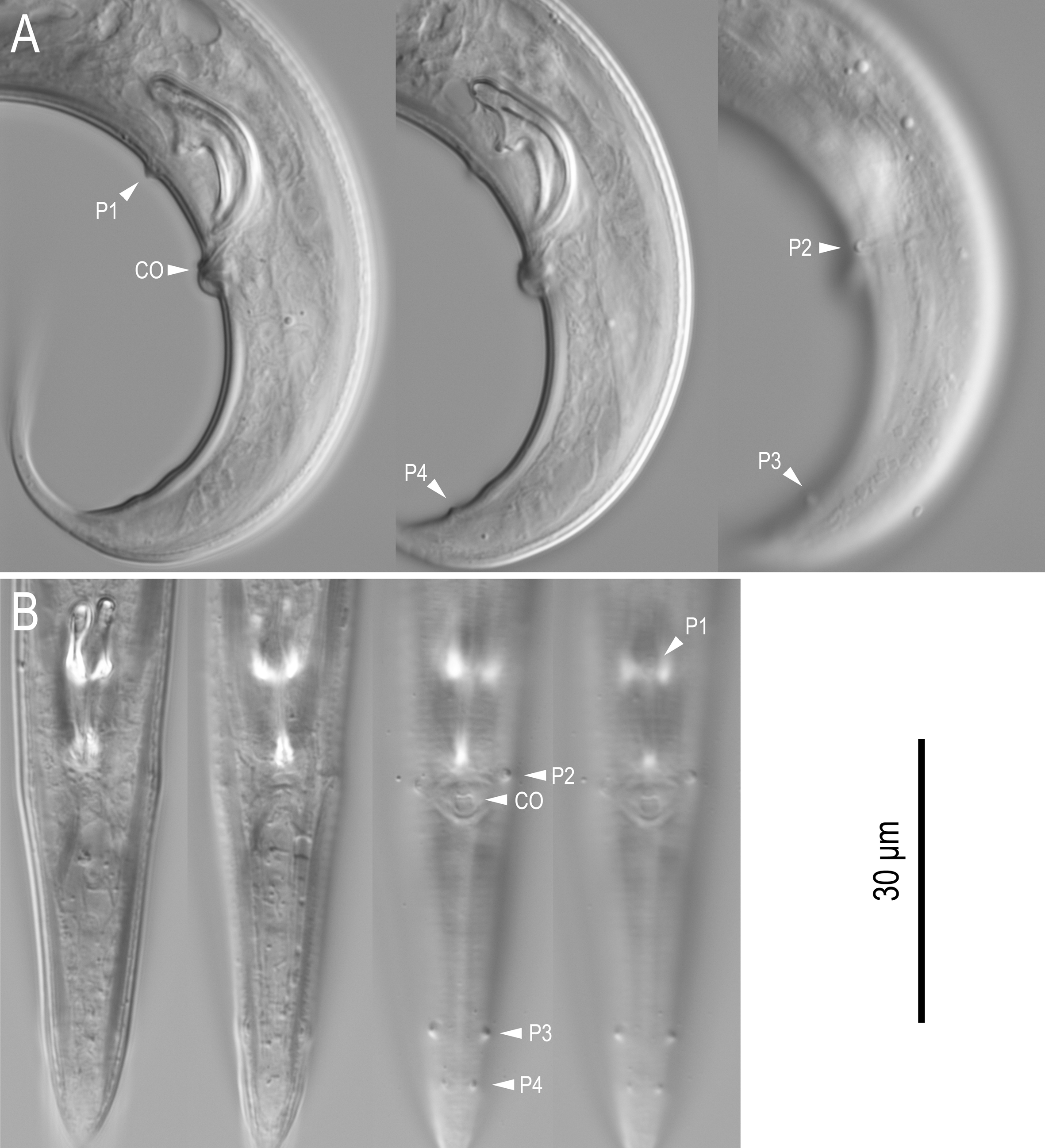

Supplement: S2 Fig — A: Lateral view in three different focal planes; B: Ventral view in four different focal planes. Abbreviations are as follows: CO = cloacal opening; P + number = genital papillae. (TIF) [file pone.0244653.s002.tif]

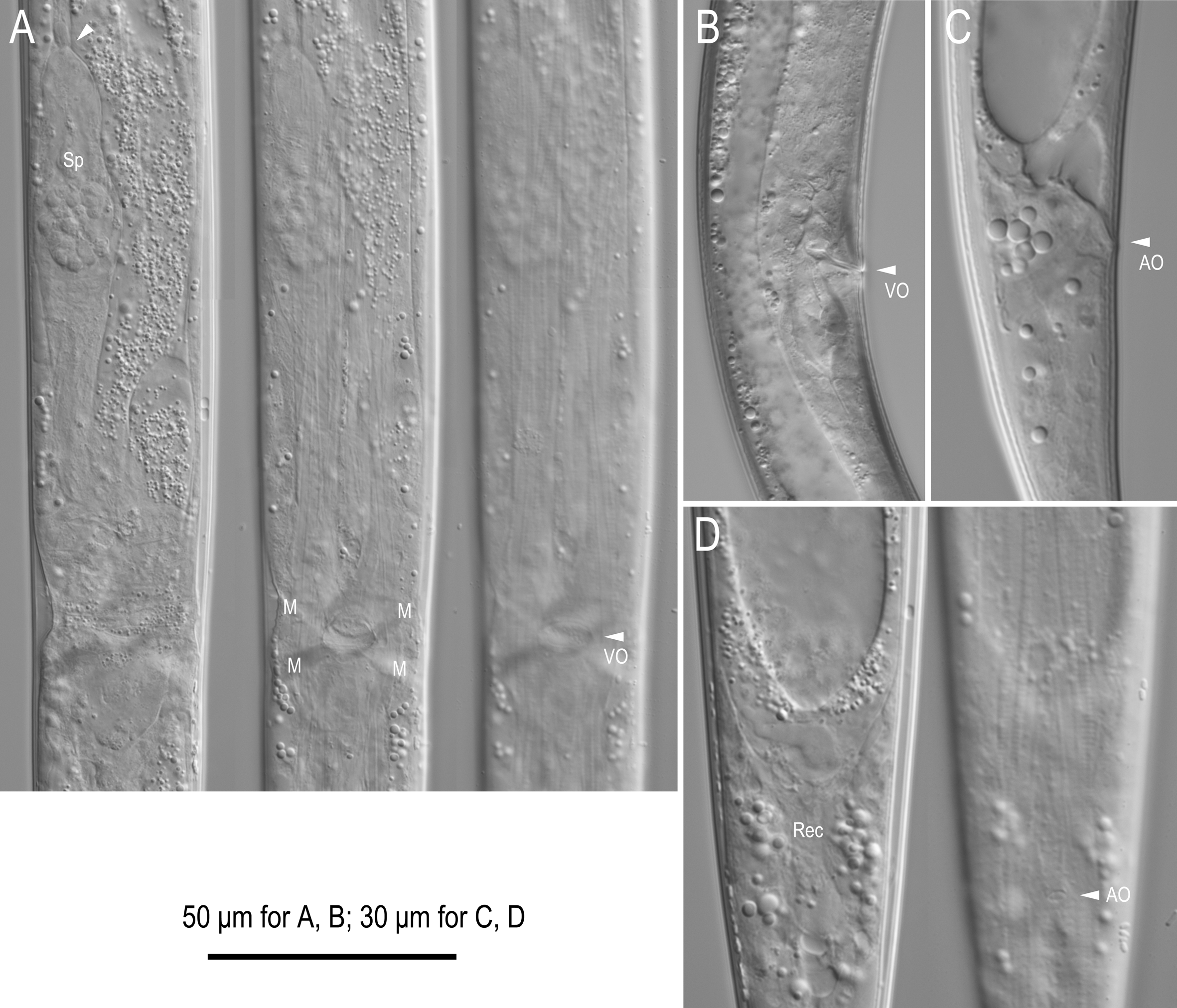

Supplement: S3 Fig — A: Ventral view of vulval region in three different focal planes; B: Lateral view of vulval region in two different focal planes; C: Ventral view of rectal-anal region in two different focal planes. Abbreviations are as follows: Sp = spermatheca where anterior end is indicated with an arrowhead; M: vulval muscle; VO = vulval opening; AO = anal opening; Rec = rectum. (TIF) [file pone.0244653.s003.tif]

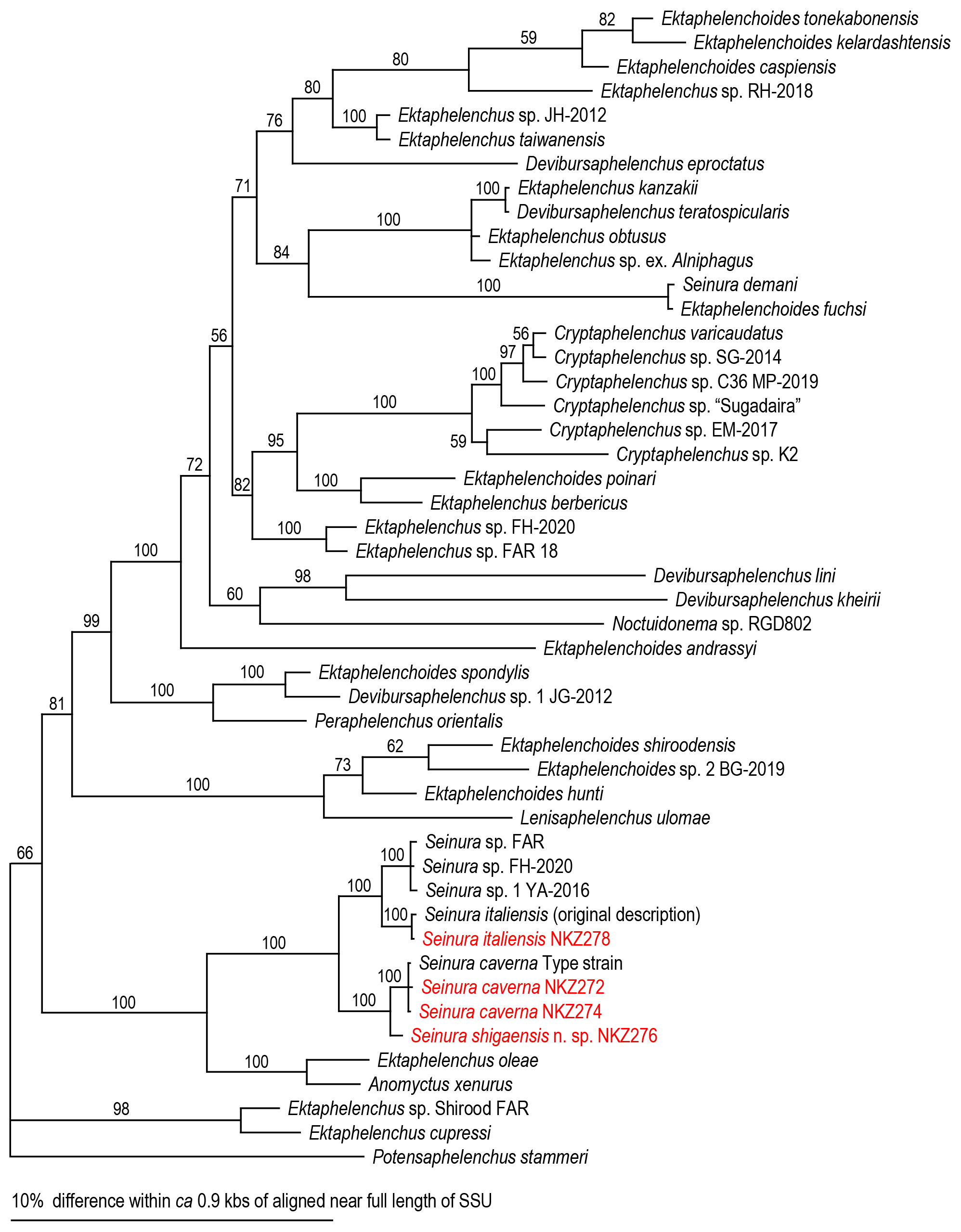

Supplement: S5 Fig — The substitution model and analytical parameters are same as the combined tree (Fig 1). Posterior probability support exceeding 50% are presented on appropriate clades. (TIF) [file pone.0244653.s005.tif]

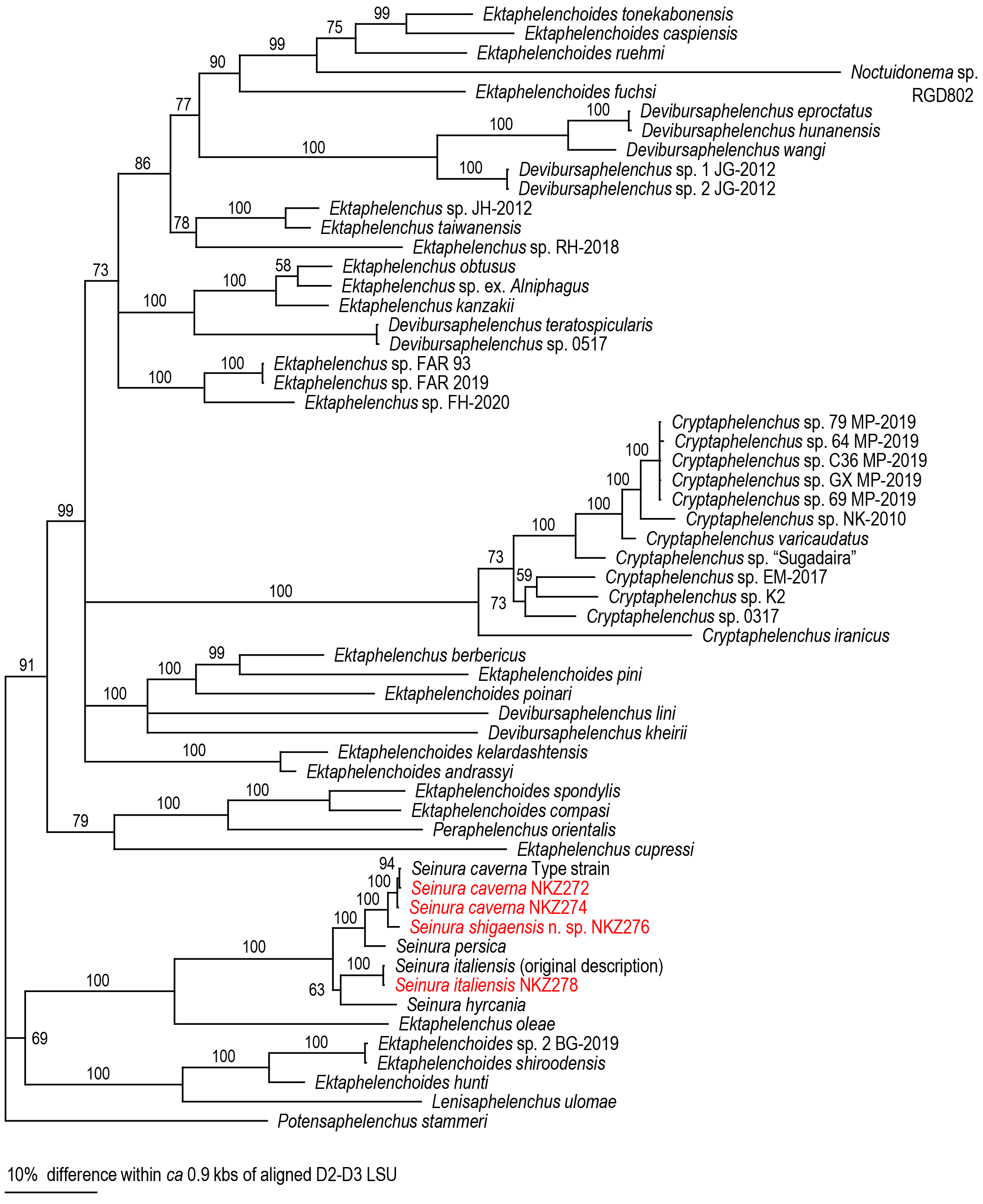

Supplement: S6 Fig — The substitution model and analytical parameters are same as the combined tree (Fig 1). Posterior probability support exceeding 50% are presented on appropriate clades. (TIF) [file pone.0244653.s006.tif]
