# Supplementary material for: Three Seinura species from Japan with a description of S. shigaensis n. sp. (Tylenchomorpha: Aphelenchoididae)
Source: PLoS One. 2021 Jan 6;16(1):e0244653. doi: 10.1371/journal.pone.0244653 (PMC7787460; doi:10.1371/journal.pone.0244653)
Supplement: S1 Table — (DOCX) [file pone.0244653.s007.docx]

**S1 Table. The molecular sequences of clade 3 aphelenchoidid species compared in the present study.**

| Species | D2-D3 LSU | SSU |
| --- | --- | --- |
| *Potensaphelenchus stammeri* | AM396582 | AB368535 |
| *Anomyctus xenurus* | - | FJ040413 |
| *Cryptaphelenchus iranicus* | KT895255 | - |
| *Cryptaphelenchus varicaudatus* | KY828211 | KY828212 |
| *Cryptaphelenchus* sp. EM-2017 | KY385333 | KY385332 |
| *Cryptaphelenchus* sp. 0317 | MG865775 | - |
| *Cryptaphelenchus* sp. 64 MP-2019 | MN325027 | - |
| *Cryptaphelenchus* sp. 69 MP-2019 | MN325026 | - |
| *Cryptaphelenchus* sp. 79 MP-2019 | MN325028 | - |
| *Cryptaphelenchus* sp. C36 MP-2019 | MN325024 | MN325022 |
| *Cryptaphelenchus* sp. GX MP-2019 | MN325025 | - |
| *Cryptaphelenchus* sp. K2 | EU287596 | EU287588 |
| *Cryptaphelenchus* sp. NK-2010 | AB597985 | - |
| *Cryptaphelenchus* sp. SG-2014 | - | KJ705087 |
| *Cryptaphelenchus* sp. “Sugadaira” | LC597088 | |
| *Devibursaphelenchus eproctatus* | JN122009 | JN122012 |
| *Devibursaphelenchus hunanensis* | FJ768945 | - |
| *Devibursaphelenchus kheirii* | KJ462485 | - |
| *Devibursaphelenchus lini* | FJ768944 | FJ768946 |
| *Devibursaphelenchus teratospicularis* | MH595683 | MH595681 |
| *Devibursaphelenchus wangi* | GQ903770 |  |
| *Devibursaphelenchus* sp. 0517 | MG865778 |  |
| *Devibursaphelenchus* sp. 1 JG-2012 | KC154094 | KC154092 |
| *Devibursaphelenchus* sp. 2 JG-2012 | KF452046 | - |
| *Ektaphelenchoides andrassyi* | KM272330 | - |
| *Ektaphelenchoides caspiensis* | KM272329 | - |
| *Ektaphelenchoides compasi* | DQ257625 | - |
| *Ektaphelenchoides fuchsi* | KJ190832 | - |
| *Ektaphelenchoides hunti* | JN714466 | - |
| *Ektaphelenchoides kelardashtensis* | JQ446374 | - |
| *Ektaphelenchoides pini* | DQ257623 | - |
| *Ektaphelenchoides poinari* | KP313828 | KC881252 |
| *Ektaphelenchoides ruehmi* | KF509853 | - |
| *Ektaphelenchoides spondylis* | AB434934 | AB849952 |
| *Ektaphelenchoides tonekabonensis* | MH751557 | MH751558 |
| *Ektaphelenchus berbericus* | KU373124 | KU373123 |
| *Ektaphelenchus cupressi* | MH544686 | MH544685 |
| *Ektaphelenchus kanzakii* | MH595685 | MH595682 |
| *Ektaphelenchus obtusus* | AB368533 | AB368532 |
| *Ektaphelenchus oleae* | KM370169 | KX621006 |
| *Ektaphelenchus taiwanensis* | JX154587 | JX154588 |
| *Ektaphelenchus* sp. JH-2012 | JX979196 | JX979194 |
| *Ektaphelenchus* sp. RH-2018 | MF043924 | MF043925 |
| *Ektaphelenchus* sp. ex. *Alniphagus* | LC414972 | LC414972 |
| *Lenisaphelenchus ulomae* | LC521308 | LC521309 |
| *Noctuidonema* sp. RGD802 | AB470970 | AB470969 |
| *Peraphelenchus orientalis* | AB786909 | AB786908 |
| *Seinura caverna* Type strain | LC414971 | |
| *Seinura caverna* NKZ272 | LC596442 | |
| *Seinura caverna* NKZ274 | LC596443 | |
| *Seinura demani* | - | FJ969140 |
| *Seinura hyrcania* | KT354242 | MN450156 |
| *Seinura hyrcania* Golestan | KT355495 | - |
| *Seinura* *italiensis* Italian population | MN428136 | MN428135 |
| *Seinura* *italiensis* NKZ278 | LC596441 | |
| *Seinura persica* Fars | KT355496 | - |
| *Seinura* sp. 1 YA-2016 | - | KU991832 |
| *Seinura* *shigaensis* n. sp. | LC596440 | |

*Potensaphelenchus stammeri* belonging to clade 4 served as outgroup species.
