# Supplementary material for: Three Seinura species from Japan with a description of S. shigaensis n. sp. (Tylenchomorpha: Aphelenchoididae)
Source: PLoS One. 2021 Jan 6;16(1):e0244653. doi: 10.1371/journal.pone.0244653 (PMC7787460; doi:10.1371/journal.pone.0244653)
Supplement: S2 Table — (DOCX) [file pone.0244653.s008.docx]

**S2 Table. Morphometric values for two populations of *Seinura* *italiensis­*.**

|  | Japanese population  (Present study) | | Italian population  (Gu *et al.*, 2018) | |
| --- | --- | --- | --- | --- |
|  | Male | Female | Male | Female |
| n | 10 | 10 | 15 | 15 |
| L | 684 ± 32  (629-735) | 817 ± 51  (758-912) | 477 ± 41  (407-565) | 522 ± 36  (469-590) |
| a | 35.6 ± 1.8  (32.8-38.8) | 31.4 ± 2.6  (28.0-35.5) | 31.4 ± 2.1  (28.6-36.3) | 29.6 ± 1.6  (26.7-33.7) |
| b | 9.1 ± 1.2  (8.2-12.4) | 9.7 ± 0.4  (9.0-10.2) | 6.7 ± 0.5  (5.9-7.6) | 7.0 ± 0.4  (6.3-7.5) |
| c | 11.8 ± 0.5  (11.1-12.7) | 7.5 ± 0.5  (6.8-8.3) | 12.5 ± 1.2  (10.5-14.6) | 9.1 ± 1.3  (7.5-12.5) |
| c' | 4.5 ± 0.2  (4.2-4.9) | 7.8 ± 0.5  (7.2-8.5) | 3.6 ± 0.3  (3.1-4.2) | 5.6 ± 0.7  (3.9-6.5) |
| T or V | 47.5 ± 7.5  (36.5-63.6) | 70.2 ± 1.2  (68.4-71.2) | 43.2 ± 11.2  (28.1-73.9) | 72.2 ± 1.5  (69.7-75.3) |
| M | 38.9 ± 1.8  (35.6-41.3) | 36.6 ± 3.2  (29.8-41.2) | *ca* 40 | |
| Maximum body diam. | 19.2 ± 1.0  (17.3-20.4) | 26.2 ± 2.6  (21.9-30.0) | 15.2 ± 1.2  (13.3-16.9) | 17.7 ± 1.4  (15.7-20.9) |
| Lip diam. | 8.7 ± 0.4  (8.2-9.2) | 9.5 ± 0.4  (9.2-10.2) | 6.8 ± 0.6  (5.8-8.0) | 7.3 ± 0.4  (6.6-8.1) |
| Lip height | 3.8 ± 0.4  (3.1-4.1) | 4.0 ± 0.4  (3.6-4.6) | 3.0 ± 0.3  (2.8-3.5) | 3.4 ± 0.4  (2.8-4.1) |
| Lip height/diam. | 2.3 ± 0.2  (2.0-2.7) | 2.4 ± 0.2  (2.1-2.7) | *ca* 2.0 | |
| Stylet conus | 9.1 ± 0.5  (8.2-9.7) | 10.0 ± 0.5  (8.7-10.7) | Not given | |
| Stylet length | 23.5 ± 1.2  (22.5-26.5) | 27.4 ± 1.4  (26.0-30.6) | 17.4 ± 1.5  (14.5-20.1) | 20.6 ± 1.6  (18.3-23.6) |
| Metacorpus diam. | 11.6 ± 0.5  (11.2-12.2) | 13.5 ± 0.8  (12.2-14.3) | 8.7 ± 0.7  (7.5-10.2) | 10.2 ± 0.6  (9.4-11.8) |
| Metacorpus length | 20.3 ± 1.0  (19.4-22.4) | 22.9 ± 1.4  (20.4-25.0) | 15.4 ± 1.0  (13.8-17.4) | 17.8 ± 1.5  (14.8-21.0) |
| Metacorpus length/diam. ratio | 1.7 ± 0.1  (1.6-1.9) | 1.7 ± 0.1  (1.6-1.8) | 1.8 ± 0.1  (1.6-2.0) | 1.7 ± 0.1  (1.5-1.9) |
| Secretory-excretory pore from anterior end | 74 ± 2.7  (71-79) | 78 ± 5.2  (67-85) | 66 ± 3.8  (55-69) | 68 ± 4.9  (57-74) |
| Nerve ring from anterior end | 84 ± 3.4  (79-90) | 87 ± 3.6  (83-95) | Not given | |
| Hemizonid from anterior end | 97 ± 4.4  (91-103) | 103 ± 4.2  (96-110) | 82 ± 4.5  (73-90) | 84 ± 4.0  (78-92) |
| Testis or ovary length | 323 ± 41  (260-400) | 354 ± 36  (304-411) | 206 ± 55  (143-340) | 180 ± 36  (113-232) |
| Reflexed part of gonad | 58, 60  (n = 2) | 0 | 0 | 0 |
| Cloacal or anal body diam. | 13.0 ± 0.4  (12.2-13.8) | 14.1 ± 1.1  (12.2-15.3) | 10.7 ± 0.6  (9.6-11.6) | 10.5 ± 0.7  (46.3-72.0) |
| Tail length | 58 ± 2.1  (53-60) | 109 ± 4.9  (103-118) | 39 ± 4.5  (29-45) | 58 ± 6.8  (44-72) |
| *Vas deferens* length | 90 ± 8.1  (78-104) | - | Not given | - |
| % of *vas deference* to total gonad | 28.1 ± 3.8  (22.4-34.5) | - | Not given | - |
| Spicule (chord) | 17.5 ± 1.0  (15.8-19.4) | - | 14.1 ± 0.8  (12.6-15.0) | - |
| Spicule (curved along median line) | 16.5 ± 0.8  (15.8-17.9) | - | 14.5 ± 1.0  (12.7-15.8) | - |
| Vulval body diam. | - | 23.0 ± 1.9  (19.9-26.5) | - | Not given |
| Vulva-anus distance | - | 133 ± 7.5  (121-146) | - | 87 ± 7.4  (74-96) |
| Post-uterine sac (PUS) length | - | 68 ± 5.2  (62-78) | - | 59 ± 5.6  (51-69) |
| PUS % to vulva-anus distance | - | 50.7 ± 2.6  (48.5-55.5) | - | 68.2 ± 7.7  (58.1-85.3) |
| PUS / vulval body diam. | - | 3.0 ± 0.2  (2.7-3.2) | - | 2-4 |

All measurements are in μm and in the form: mean ± s.d. (range).
